# Prevalence of HIV and other sexually transmitted infections and their association with sexual practices and substance use among 2238 MSM in Lebanon

**DOI:** 10.1038/s41598-019-51688-7

**Published:** 2019-10-22

**Authors:** Ayman Assi, Sara Abu Zaki, Jade Ghosn, Nizar Kinge, Jihane Naous, Antoine Ghanem, Diana Abou Abbas, Ziad Bakouny, Georges Azzi, Roland Tomb

**Affiliations:** 1 Marsa Sexual Health Center, Beirut, Lebanon; 2 Faculty of Medicine, University of Saint-Joseph in Beirut, Beirut, Lebanon; 3 Assistance Publique–Hôpitaux de Paris, Service des Maladies Infectieuses et Tropicales, Groupe Hospitalier Paris Nord Val de Seine, site Bichat-Claude Bernard, Paris, France; 4 Université Paris Diderot, INSERM UMR 1137 IAME, PRES Sorbonne Paris Cité, Paris, France

**Keywords:** HIV infections, HIV infections

## Abstract

UNAIDS report documents 95% increase in new HIV infections among key populations in Eastern Europe and Middle East and North Africa region. Data on HIV and STIs among MSM in Lebanon is still scarce. Therefore, the aim was to assess prevalence of HIV and sexually transmitted infections (STIs) among men who have sex with men (MSM) in Lebanon and associations with sexual practices and substance-use. 2238 MSM attended a sexual health clinic in Lebanon between 2015–2018. Demographics, substance-use and sexual practices were collected. Attendees tested for HIV and other STIs. HIV infection was diagnosed in 5.6% of the sample. Only 19% received sexual health education from reliable sources (school/university/healthcare workers), 78% reported having multiple partners in the past three months (2–5 partners: 58%, 6+: 20%) and 67% reported inconsistent condom-use. Moreover, 40% of HIV + cases were returning attendees who already received information about condom-use. Additionally, having only a school level education (11%) increases the odds of having inconsistent condom-use with casual partners (adj.OR:1.9, p < 0.001). The results reflect the urgent need for: (1) accurate and comprehensive sexual health and harm reduction education and promotion in Lebanon; (2) making pre-exposure prophylaxis available for free to key populations to contain the epidemics at an early stage.

## Introduction

The UNAIDS shared in July 2018 the alarming numbers of new human immunodeficiency virus (HIV) infections in key populations: 47% increase globally, 95% increase in Eastern Europe and Central Asia as well as in the Middle East and North Africa (MENA)^1^. The risk of acquiring HIV is 27 times higher among men who have sex with men (MSM), compared to men who have sex with women globally^1^. With the emergence of more data on HIV in the MENA region, hidden epidemics among key population groups, such as MSM, have not only been identified across the region but may also be growing in size^2^, mirroring the latest findings of UNAIDS. While a number of studies have found high levels of risky sexual practices and substance use among MSM around the world^3,4^, there are insufficient data in the MENA region in general and in Lebanon in particular. Additionally, a global study conducted by the World Health Organization (WHO) in 2016 emphasized the importance of increasing research efforts on sexually transmitted infections (STIs) given the high rates of infections globally^5^.

Lebanon, a Middle Eastern country, remains conservative especially with topics related to sex and sexuality. Penal code 534 criminalizes sexual acts that are “against nature”; and is commonly used against the Lesbian, Gay, Bisexual, Transgender, and Queer (LGBTQ) community and particularly against MSM^6^. Substance use is also highly stigmatized and criminalized in Lebanon^6^. These punitive laws put additional strains on people living in Lebanon and especially those who are LGBTQ, thus limiting their access to necessary sexual health and harm reduction services^7,8^. The National AIDS Program (NAP) of the Ministry of Public Health in Lebanon (MOPH) reported a cumulative of 2366 people living with HIV (PLHIV) in Lebanon by the end of 2018^9^. Few studies have investigated HIV prevalence among MSM in Lebanon^10–12^, their sexual practices^10^ or condom-use^13^. Furthermore, while a strong association between substance use and risky sexual practices was reported among the Lebanese youth^14^, this was not investigated among MSM specifically. A comprehensive evaluation of the relationship between HIV and STIs prevalence, sexual practices and substance use among MSM in Lebanon has never been addressed.

Thus, the aim of this study was to assess the associations of STIs, including HIV, with risky sexual practices and substance use among MSM attending a sexual health clinic in Lebanon.

## Methods

### Sample

Data were collected from individuals voluntarily attending Marsa sexual health center (a non-governmental organization) located in Beirut, Lebanon between January 1^st^, 2015 and December 31^st^, 2018 for voluntary counseling and testing (VCT) and/or medical consultation for symptoms related to STIs.

Healthcare professionals administered an anonymous questionnaire for each attendee prior to undergoing medical testing and/or medical consultation, and a file number was delivered to each attendee. Data from the questionnaire, testing and medical consultation were saved digitally under each file number and could only be viewed by selected health professionals at the clinic who had signed a confidentiality agreement. To ensure the attendees’ anonymity, a verbal consent was obtained prior to the administered questionnaire. Attendees who did not give verbal consent could opt-out of the questionnaire administered by the health professional. Those who opted out of answering the questionnaire still received testing services. Ethical considerations, including anonymity and consent, were approved by the IRB office of our academic institution (USJ2016-77). The methods were carried out in accordance with the relevant guidelines and regulations.

The inclusion criterion was being a cisgender man having sex with men. The questionnaire included sections on demographics, substance use, sexual practices and previous unprotected sexual exposures. Age was categorized into the following groups: <20, 20–24, 25–29, 30–34, 35–39, ≥40 years old. Attendees were also asked about sources of sexual health education. The following sources were considered as reliable: school, university and/or healthcare providers; the following sources were considered as unreliable: friends/peers, sexual partners, internet/pornography; and various sources were those who reported both (reliable and unreliable sources). Attendees were asked about substance-use: smoking, alcohol, and recreational drugs such as cannabis, cocaine, muscle relaxant (poppers), ecstasy, GHB (Gamma-HydroxyButyrate), MDMA (3,4-MethyleneDioxyMethAmphetamine), heroine, ketamine. The term recreational drug-use refers to the use of a psychoactive drug to induce an altered state of consciousness for pleasure. Attendees were also asked about condom-use during penetrative sex with exclusive partners, regular partners (frequent but not exclusive), and casual partners (one-time partners). The detailed collected data were displayed in Table 1.

**Table 1:** Results of demographics, substance use, sexual practices and sexual exposures in 2238 MSM in Lebanon.

	Parameter		Missing values	Variables	Reported	Percentage (based on those who answered)
Demographics	Age		0	<20 years	84	4%
				20–24 years	731	33%
				25–29 years	725	32%
				30–34 years	378	17%
				35–39 years	205	9%
				≥40 years	115	5%
	Highest level of education		0	University	1988	89%
				School	250	11%
	Sources of sexual education		0	Reliable Sources	429	19%
				Various (both reliable and unreliable sources)	663	30%
				Unreliable Sources	1146	51%
	Occupation		0	Employed	1014	45%
				Student	1052	47%
				Jobless	172	8%
	Nationality		0	Lebanese	1905	85%
				Syrian	120	5%
				Palestenian	40	2%
				Other	173	8%
Substance use	Cigarette smoking		0	Does not smoke	1463	65%
				Smokes	775	35%
	Recreational drug use		0	Does not use	1600	71%
				Uses	638	29%
		MSM who reported mixing drugs	163	No	387	81%
				Yes	88	19%
		MSM who reported mixing drugs with alcohol	162	No	304	59%
				Yes	209	41%
		MSM who reported using drugs during their first time they had sex	357	No	269	96%
				Yes	12	4%
		MSM who reported using drugs during the last time they had sex	338	No	229	76%
				Yes	71	24%
		MSM who associate drug use with sex	102	No	318	59%
				Yes	218	41%
	Alcohol consumption		0	Does not consume alcohol	459	21%
				Consumes alcohol	1779	79%
		MSM who associate alcohol with sex	580	No	706	59%
				Yes	493	41%
Sexual practices	Condom-use with partner(s)		0	Inconsistant condom use with regular partner	239	11%
				Inconsistant condom use with casual partner	1040	46%
				Inconsistant condom use with exclusive Partner	220	10%
				Always uses condoms	739	33%
	Reason for testing		0	Condomless sex or has symptom(s) of STI	1545	69%
				Regular check-up	693	31%
	Lubricant-use during penetrative sex		0	Always uses	718	32%
				Inconsistant use	1520	68%
	Reasons for not using a condomNumber of sexual partners in last 3 months		749	Trusts Partner	537	36%
				Heat of the moment	288	19%
				Was under the influence of a substance	280	19%
				Cannot negotiate condoms with partner	52	3%
				Does not like using condoms	81	5%
				Condom was not available	66	4%
				Has misinformation about condom-use	185	12%
			0	0 to 1	485	22%
				2 to 5	1306	58%
				6 to 10	247	11%
				≥11	200	9%
Sexual exposure	Oral sex exposure		0	Always used a condom	30	1%
				Unprotected received	93	4%
in last 3 months				Unprotected performed	120	5%
				Unprotected both	1995	89%
	Anal sex exposure		0	Always used a condom	1049	47%
				Unprotected insertive	350	16%
				Unprotected receptive	410	18%
				Unprotected both	429	19%

### Collected data

Attendees who underwent VCT were given the option of being tested for viral Hepatitis B (HBV), viral Hepatitis C (HCV), syphilis (at subsidized prices) and/or HIV (for free) using rapid tests (Abon®), and the results were collected. The HIV kit was a third-generation test. Individuals who were in their serological window period after having a risk of sexual exposure were asked to do the test after completion of the window period. The latest result was included in this study.

Prevalence of HIV was reported among new and returning attendees. Some of the rapid tests were not consistently available throughout the duration of the study, specifically for HCV. Positive results were complemented by laboratory testing for confirmation (Elisa 4G and Western Blot for HIV, VDRL for Syphilis, DNA/RNA PCR for Hepatitis B/C). Positive cases were immediately referred to the clinic’s physicians. Those who tested positive were not aware of their status prior to the test.

Symptoms indicative of other STIs were documented through medical consultations. Although medical laboratory tests were provided at subsidized cost, very few attendees chose to undergo these tests due to financial constraints. Therefore, treatment relied on symptomatic diagnosis.

The explored STIs were Neisseria gonorrhea Ng/Chlamydia trachomatis Ct (presence of discharge and burning sensation during urination, and/or diagnosed biologically by Multiplex PCR test), Human Papilloma Virus (HPV, presence of genital, anal, oral warts and/or diagnosed by histology), Herpes Simplex Virus (HSV, presence of herpes zoster), scabies (presence of scabies rash), and pubic lice (presence of lice in pubic area). Medical laboratory tests were based on anal, oral or intra-uretral swabs, urine sample, and/or blood sample.

### Statistics

Prevalence of HIV, HBV, HCV, Syphilis, Ng/Ct, HPV, pubic lice and coinfections were reported. In order to predict possible determinants of testing positive for any STI, a univariate analysis (simple binomial regression) followed by a multivariate analysis (stepwise binomial regression models) were computed. The outcome variables of HIV, HBV, HCV and Syphilis were considered as dependent variables, while independent variables were all variables listed in the following categories: demographics, substance use, sexual practices, unprotected sexual exposures in the past three months.

In order to explore possible factors linked to condom and recreational drug use, a univariate analysis (simple binomial regression) followed by a multivariate analysis (stepwise binomial regression models) were performed. The dependent variable was either condom-use or recreational drug use, while the independent variables were variables listed in the following categories: demographics, substance use, sexual practices, and sexual exposures.

Only variables with significant correlations in the univariate analysis were included in the multivariate analysis. Adjusted Odds Ratios (Adj. OR) with their respective confidence intervals (CI) were reported for each model. The level of significance was set at 0.05. Statistics were performed under Xlstat^®^ (version 19.02, Addinsoft, Paris, France) and SPSS^®^ (version 20, IBM, Armonk, NY, USA).

## Results

A total of 2238 MSM attendees presented to Marsa sexual health clinic between the 1^st^ of January 2015 and the 31^st^ of December 2018. From this sample, 1326 subjects attended the clinic only once, whereas 912 (41%) attended the clinic several times during the period of the study. In total, 1477 (66%) of the sample where residing in Beirut. All 2238 underwent VCT, 502 also underwent medical consultations.

### Population characteristics

The age of the MSM sample ranged between 15 and 69 years old, with a median of 26 years (1^st^ quartile: 23; 3^rd^ quartile: 31). The results of demographics, substance use, sexual practices, and sexual exposures were displayed in Table 1.

### Prevalence of STIs

Prevalence of HIV (5.6%), HPV (41.0%;), and other STIs were displayed in Table 2. From the 119 attendees who tested positive for HIV (5.6%), 48 were returning clients (40%). Prevalence of co-infections was as follows: HIV and Syphilis in three cases; HIV and HPV in 18 cases; HIV and Ng/Ct in four cases; HIV and HCV in two cases.

**Table 2:** Prevalence of HIV and other STIs among MSM in Lebanon.

	Sexually Transmitted Infections	Total tested	Total positive	Percentages
Diagnosis through rapid test	HIV	2126	119	5.6%
	Hepatitis B	1816	9	0.5%
	Hepatitis C	758	4	0.5%
	Syphilis	1429	43	3%
Diagnosis based on symptoms	Neisseria Gonorrhea and/or Chlamydia Trachomatis	502	88	17.5%
	Human Papilloma Virus	502	206	41%
	Pubic Lice	502	15	3%
	Herpes Simplex Virus	502	11	2%
	Scabies	502	7	1.4%

Statistical models were restricted to HIV and Syphilis due to the low prevalence of HBV and HCV. The multivariate analysis showed that:

1. HIV status was found to be significantly related to anal sex intercourse only (Table 3).

**Table 3:** Associations between HIV/Syphilis status and demographics, substance use, sexual practices and sexual exposures in MSM in Lebanon.

		HIV		Univariate analysis		Multivariate analysis			Syphilis		Univariate analysis		Multivariate analysis		
	Co-variable	Number of +cases	%	Crude OR	p-value	Adjusted OR	Confidence Interval	p-value	Number of + cases	%	Crude OR	p-value	Adjusted OR	Confidence Interval	p-value
Age	<20 years	3	3%	1	0.97				1	2%	0.3	0.33	0.3		0.33
	20–24 years	29	24%	1.1	0.8				6	14%	0.2	0.02	**0.2**	**[0.064–0.827]**	**0.02**
	25–29 years	41	34%	1.6	0.34				8	19%	0.3	0.05	0.3		0.05
	30–34 years	31	26%	2.4	0.09				13	30%	0.9	0.98	0.9		0.99
	35–39 years	11	9%	1.5	0.44				11	26%	1.5	0.44	1.5		0.44
	≥40 years	4	3%	ref					4	9%	ref				
Nationality	Lebanese	101	85%	1.5	0.3				37	86%	1.1	0.84			
	Syrian	10	8%	2.5	0.08				3	7%	1.4	0.65			
	Palestenian	2	2%	1.4	0.64				0	0%	0	0.99			
	Other	6	5%	ref					3	7%	ref				
Source of sexual education	Reliable	20	17%	ref					7	16%	ref				
	Various	40	34%	1.3	0.69				12	28%	1.2	0.55			
	Unreliable	59	50%	1.1	0.33				24	56%	1.1	0.82			
Highest level of education	University	99	83%	ref					35	81%	ref				
	School	20	17%	**1.6**	**0.04**				8	19%	1.8	0.12			
Occupation	Employed	56	47%	ref					23	53%	ref				
	Student	51	43%	0.8	0.48				16	37%	0.6	0.21			
	Jobless	12	10%	1.2	0.44				4	9%	1	0.96			
Recreational drug use	Does not use	77	65%	0.7	0.09				27	63%	0.6	0.2			
	Uses	42	35%	ref					16	37%	ref				
Cigarette smoking	Does not smoke	76	64%	ref					27	63%	ref				
	Smokes	43	36%	1.2	0.21				16	37%	1.3	0.31			
Alcohol consumption	Does not consume	28	24%	ref					11	26%	ref				
	Consumes	91	76%	0.8	0.4				32	74%	0.7	0.4			
Condom-use with partner(s)	Always uses condoms	33	28%	ref					11	26%	ref				
	Inconsistant condom use with exclusive partner	11	9%	1.1	0.74				4	9%	1.2	0.73			
	Inconsistant condom use with regular partner	15	13%	1.4	0.26				4	9%	1.1	0.84			
	Inconsistant condom use with casual partner	60	50%	1.3	0.22				24	56%	1.5	0.22			
Reason for testing	Regular check-up	41	34%	ref					11	26%	ref				
	Condomless sex or has symptom(s) of STI	78	66%	0.8	0.39				32	74%	1.4	0.27			
Number of sexual partners in last 3 months	0 to 1	21	18%	ref					6	14%	ref				
	2 to 5	73	61%	1.3	0.51				22	51%	1.3	0.49			
	6 to 10	14	12%	1.3	0.42				7	16%	2.3	0.13			
	≥11	11	9%	1.2	0.28				8	19%	**3.3**	**0.02**			
Oral sex exposure in last 3 months	Always used a condom	4	3%	ref					1	2%	ref				
	Did not consistently use a condom	115	97%	0.35	0.06				42	98%	0.5	0.57			
Anal sex exposre in last 3 months	Always used a condom	27	23%	ref					9	21%	ref				
	Did not consistently use a condom	92	77%	**1.7**	**0.03**	**1.7**	**[1.1–2.4]**	**0.03**	34	79%	0.5	0.13
2. Syphilis status was found to be significantly related with age only (Table 3).

### Condom-use and other sexual practices

Inconsistent condom-use during anal sex were reported by 67% (Table 1). In addition, 78% had more than one sexual partner in the last three months. Having had condomless sex or having symptoms of an STI were the primary reasons for attending (69%). Among the reasons for the absence of condom-use, 19% were under the influence of a substance (alcohol in 71%, recreational drug in 14% and mixing alcohol with drugs in 15%). The majority reported unprotected oral and anal sex exposures in the past three months (99% and 53% respectively).

The mutinomial regression model showed that: 1) those who reported having only a school level education were found to be at greater odds of having inconsistent condom-use with casual partners compared to those who reported having a university level education (adj. OR: 1.9); 2) those who received sexual health education from unreliable sources were at higher odds of having inconsistent condom-use with casual partners compared to those who received it from reliable sources (adj. OR: 1.6); 3) Those who reported having condomless anal sex in the past 3 months were more likely to have inconsistent condom-use with casual partners compared to those who did not have condomless anal sex (adj. OR: 7.6; Table 4).

**Table 4:** Determinants of condom-use with casual partner(s) and substance use among demographics, sexual practices and sexual exposures in MSM in Lebanon.

		Condom Use: Inconsistent condom-use with casual partners		Univariate analysis		Multivariate analysis			Recreational Drug-use		Univariate analysis		Multivariate analysis		
	Co-variable	N	%	Crude OR	p-value	Adjusted OR	Confidence Interval	p-value	N	%	Crude OR	p-value	Adjusted OR	Confidence Interval	p-value
Age	<20 years	42	4%	1.3	0.39				15	2%	0.7	0.33	0.6		0.24
	20–24 years	350	34%	0.9	0.93				206	32%	1.2	0.29	1.1		0.58
	25–29 years	323	31%	0.9	0.81				216	34%	1.4	0.16	1.3		0.33
	30–34 years	180	17%	1	0.95				128	20%	**1**.**6**	<**0**.**001**	**1**.**7**	**[1**.**1–2**.**7]**	<**0**.**001**
	35–39 years	92	9%	0.9	0.78				46	7%	0.9	0.83	0.9		0.77
	≥40 years	53	5%	ref					27	4%	ref				
Nationality	Lebanese	871	84%	0.9	0.77				553	87%	1.1	0.5			
	Syrian	66	6%	1.2	0.36				27	4%	0.8	0.42			
	Palestenian	21	2%	1.8	0.18				12	2%	1.1	0.66			
	Other	82	8%	ref					46	7%	ref				
Source of sexual education	Reliable	161	15%	ref					109	17%	ref				
	Various	296	28%	**1**.**3**	**0**.**03**	1.3		0.06	195	31%	1.2	0.15			
	Unreliable	583	56%	**1**.**7**	<**0**.**001**	**1**.**6**	**[1**.**2–2]**	**0**.**002**	334	52%	1.2	0.14			
Highest level of education	University	898	86%	ref					586	92%	ref				
	School	142	14%	**1**.**9**	<**0**.**001**	**1**.**9**	**[1**.**3–2**.**7]**	<**0**.**001**	52	8%	**0**.**62**	**0**.**004**	**0**.**62**	**[0**.**44–0**.**87]**	**0**.**015**
Occupation	Employed	443	43%	ref					272	43%	ref				
	Student	503	48%	1.1	0.17				317	50%	1.2	0.65	1.1		0.44
	Jobless	94	9%	**1**.**5**	**0**.**02**				49	8%	1.1	0.09	**1**.**3**	**[1**.**1–1**.**7]**	**0**.**004**
Recreational drug use	Does not use	744	72%	1	0.63										
	Uses	296	28%	ref					638						
Cigarette smoking	Does not smoke	332	32%	ref					326	51%	ref				
	Smokes	708	68%	1.2	0.06				312	49%	**3**.**4**	<**0**.**001**	**3**.**4**	**[2**.**7–4**.**1]**	<**0**.**001**
Alcohol Consumption	Does not consume	234	23%	ref					93	15%	ref				
	Consumes	806	78%	0.7	0.006				545	85%	**1**.**7**	<**0**.**001**	**1**.**7**	**[1**.**3–2**.**2]**	<**0**.**001**
Condom-use with partner(s)	Always uses condoms								218	34%	ref				
	Inconsistant condom use with exclusive partner								55	9%	0.79	0.19			
	Inconsistant condom use with regular partner								69	11%	0.97	0.85			
	Inconsistant condom use with casual partner	1040							296	46%	0.95	0.63			
Number of sexual partners in last 3 months	0 to 1	177	17%	ref					98	15%	ref				
	2 to 5	631	61%	1.2	0.13	1.2		0.13	363	57%	**1**.**5**	<**0**.**001**	**1**.**6**	**[1**.**2–2**.**1]**	<**0**.**001**
	6 to 10	123	12%	1.2	0.32	1.2		0.2	96	15%	**2**.**5**	<**0**.**001**	**2**.**7**	**[1**.**8–3**.**8]**	<**0**.**001**
	≥11	109	10%	**1**.**5**	**0**.**03**	1.5		0.05	81	13%	**2**.**7**	<**0**.**001**	**3**	**[2–4·2]**	<**0**.**001**
Oral sex exposure in last 3 months	Always used a condom	9	1%	ref					8	1%	ref				
	Did not consistently use a condom	1031	99%	**2**.**3**	**0**.**04**				630	99%	1.1	0.82			
Anal sex exposre in last 3 months	Always used a condom	323	31%	ref					199	31%	ref				
	Did not consistently use a condom	717	69%	**7**.**7**	<**0**.**001**	**7**.**6**	**[6**.**1–9**.**5]**	<**0**.**001**	439	69%	1.02	0.82			

### Substance use

**Figure 1:**
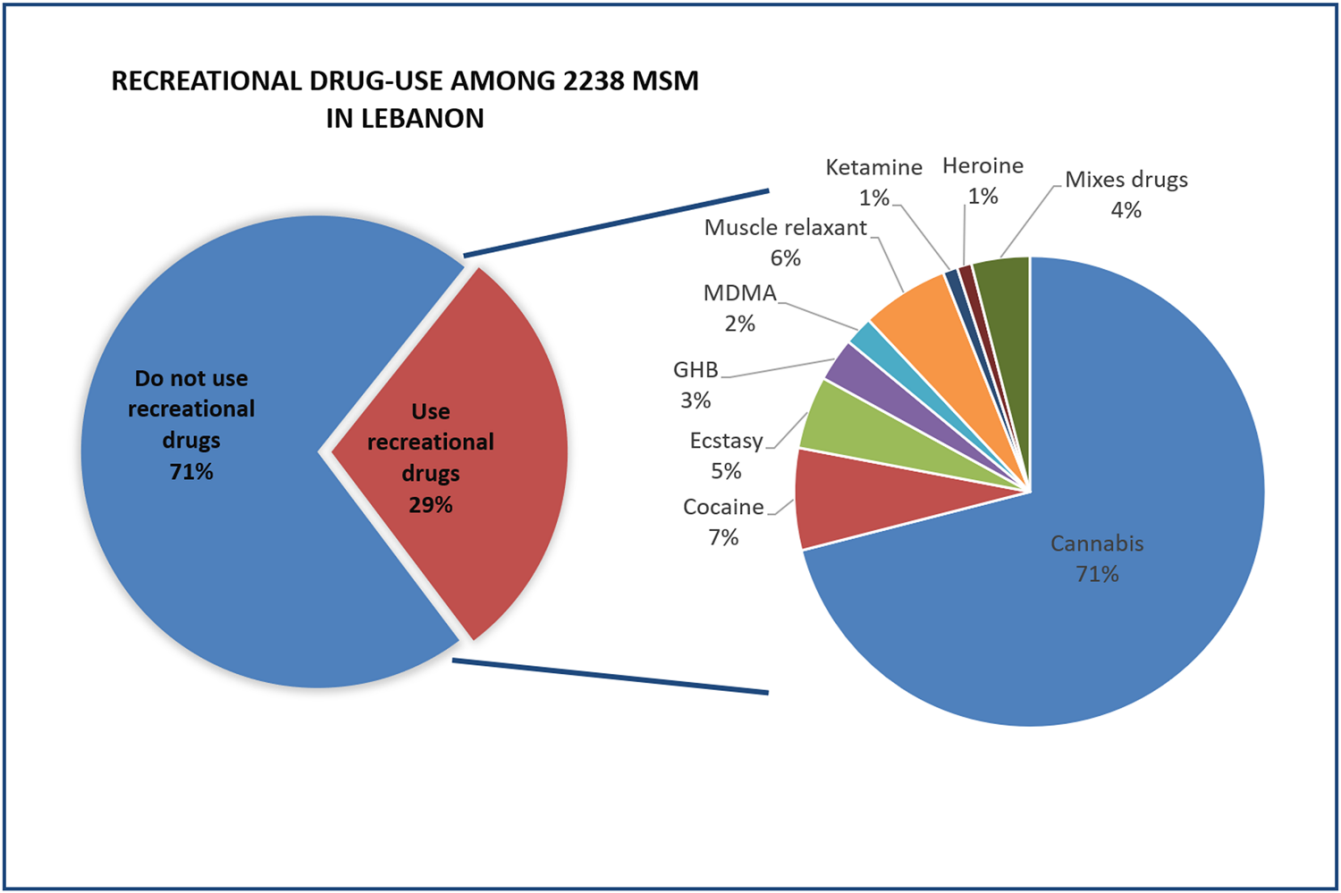
Substance use in 2238 MSM in Lebanon: recreational drug use in addition to the reported types of drugs.

Cigarette smoking was reported in 35% of the sample, while 79% reported alcohol consumption and 29% reported using recreational drugs (details in Fig. 1). The binomial regression model showed that recreational drug use was found to be significantly associated with age, level of education, number of sexual partners, occupation, alcohol and smoking status; more precisely, those who reported having multiple partners (more than 2) in the last three months were found to be at greater odds of using recreational drugs (having between two to five partners adj. OR: 1.6, having between six to ten partners adj. OR: 2.7, having more than ten partners adj. OR: 3) than those who reported having 0 to 1 partner in the last three months (more details in Table 4).

## Discussion

Sex and sexuality are highly stigmatized in the MENA region and in Lebanon, especially for those with non-normative sexual practices. UNAIDS has drawn attention to the rising number of new HIV infections in the MENA region among key affected populations such as MSM. This study assessed the prevalence of HIV and other STIs as well as sexual practices and substance use among 2238 MSM in Lebanon. The prevalence of HIV was 5.6% (40% were returning attendees) and 41% presented with genital warts.

This study was based on attendees who voluntarily came to the clinic, most of them were young and were university students (47%), or had a university level education (89%), as in previous studies based on respondent driven samples (RDS)^11^. Sexual health education is not implemented in the majority of schools and universities in Lebanon, and there is little access to accurate and comprehensive information about sexual health. This is obvious in the result obtained in this study where only 19% of the sample received sexual health education from reliable sources.

Prevalence of HIV and other STIs in this study were higher than those reported in previous studies on MSM in Lebanon^11,15^. HIV prevalence in this study was 5.6% (out of 2126 MSM who underwent HIV testing) while it was 3.7% (out of 101 MSM)^15^, and 1.5% (out of 213 MSM)^11^ in previous studies. Only one local study found a higher HIV prevalence rate of 12.3% among its sample of 292 MSM recruited through RDS. However, the authors highlighted a number of factors that may have overestimated the rate of HIV prevalence such as: 1) the majority of HIV positive cases had already known their status prior to the study and 2) 25% of the sample were Syrian individuals who have moved to Lebanon recently^12^.

Even with the relatively lower prevalence of HBV compared to other STIs among our sample, it remains necessary to promote HBV testing and vaccination to key populations and particularly MSM in order to prevent further spread of the virus. The low prevalence of HCV among the sample (0.5%) could be indicative of a low prevalence of injection drug use^16^.

No official local data exist on prevalence of Syphilis, Ng/Ct among the MSM population in Lebanon. In this study, 3% of the MSM who underwent the syphilis rapid test had positive results, which is lower than previous reports in the literature^17,18^, notably among Chinese MSM (8%) and a sample of Peruvian MSM (4.4%). The positive association between syphilis and age may be attributed to greater time for exposure.

Furthermore, 17.5% of 502 individuals who sought medical consultation were treated for Ng/Ct based on indicative symptoms, a percentage higher than rates reported in a study on a sample of MSM in Germany^19^.

Genital warts were reported among 41% of the MSM who presented for medical consultations which is greater than the only other reported prevalence of HPV among MSM in Lebanon (10% among 42 MSM)^20^.

Upon examining common sexual practices, the majority had inconsistent condom-use (67%) as previously shown^11^. Among those, 46% reported inconsistent condom-use with casual partners, whose STI status (including HIV) was likely unknown to them, which would therefore put them at an increased risk of STIs. The model run on condom-use found that those who had only a school level education and those who reported receiving sexual health education from unreliable sources (such as peers, sexual partners, internet or pornography) were at higher odds of having inconsistent condom-use. This is likely attributed to the lack of sexual health education in the Lebanese educational system, especially at schools. These results should be used to lobby governmental agencies to include a comprehensive sexual health curriculum in schools.

While data on substance use and sexual practices among MSM exist across different regions, this is the first study to explore substance use among MSM in Lebanon. The connection between alcohol use and risky sexual practices among MSM has already been reported in previous studies in Australia^21^. This was also evident in this study where 19% of the sample stated that they did not use a condom during their last sexual intercourse because they were under the influence of a substance; among those alcohol being that substance in 71%.

With nearly one third of the sample reporting recreational drug use, further exploration of the determinants of drug use was warranted. No local sources were identified that evaluated drug use among MSM in Lebanon. However, various international sources attest to the fact that MSM are more likely to use drugs than their heterosexual peers^22,23^. Previous studies showed that drug use was found to be associated with different risky sexual practices^22,23^. The latter is concurrent with findings from this study, where MSM who reported multiple sexual partners in the past month, as well as those who were smokers, were at higher odds of using recreational drugs. Additionally, 29% of those who reported not using a condom because of substance use were using recreational drugs or mixing drugs with alcohol. The latter is also known to be a common trigger for risky sexual practices among MSM in different regions^22,24^.

This study has some limitations that should be considered. The quality of sexual health education provided in schools in Lebanon might vary, which could affect the efficacy of the results related to sexual health education. Additionally, a reporting bias might be present in the analysis as the topics discussed during the administered questionnaire are of a sensitive nature and therefore individuals might have refrained from disclosing risky sexual practices due to fears of being stigmatized. However, the sexual health center, that has been active since 2011, is well trusted by the MSM community for providing quality anonymous sexual health services in a non-stigmatizing and confidential environment. This will likely reduce the risk of reporting bias. Moreover, the heterogeneity of the sample with regards to age, nationality, education level etc. may affect the generalizability of the results.

However, to the best of our knowledge, this study remains the first comprehensive study to assess different STIs, sexual practices and substance use, including the largest number of MSM in Lebanon and MENA region^25,26^.

Another possible limitation might be the use of symptomatic methods of diagnosing Ng/Ct and HPV rather than laboratory testing. This was due to the high cost of laboratory testing that most attendees could not afford. However, symptoms such as genital warts or burning sensation during urination and/or penile or anal discharge are known to be indicative of HPV or Ng/Ct, respectively. In fact, it is likely that the reported prevalence of HPV and Ng/Ct are underestimated since these infections can be asymptomatic.

A sampling bias might also exist since this sample is comprised of individuals presenting to a sexual health clinic and therefore actively seeking STI testing and/or information regarding sexual health. They are therefore more likely to be sexually active and/or to have engaged in risky sexual practices. Nevertheless, the sample included in this study represents 37% of the MSM population estimated to be residing in Beirut^27^.

The relatively high prevalence of HIV detected in this study confirms the presence of pockets of endemic transmission among MSM which were not easily detected in previous studies conducted in Lebanon using RDS. Even though evidence of its success in decreasing new infections among some MSM communities has been documented^28^, pre-exposure prophylaxis (PrEP) is still not provided by the MOPH, except for married heterosexual sero-discordant couples, and is very expensive when purchased at pharmacies. Forty percent of those tested positive for HIV were returning attendees indicating that receiving information about condom-use to prevent HIV transmission, during their previous visit(s), was not sufficient. Negotiations with the MOPH and international agencies (UNAIDS and WHO) to make PrEP available to the MSM community in Lebanon for free or at a subsidized cost should be accelerated in order to contain the epidemic at an early stage. Similarly, the high prevalence of HPV indicates an urgent need for promoting screening and prevention through provision of HPV vaccine for free or at a subsidized cost.

Finally, advocacy efforts must highlight the importance of decriminalizing homosexuality to improve MSM’s access to HIV testing and other sexual health services. This is especially important since stigma has been found to deter the success of HIV prevention programs and activities^28,29^.

## Conclusions

The results of this study portray the importance of enhancing sexual health services in Lebanon, especially with the rising prevalence of HIV and STIs locally and in the MENA region. These results are key in making a case to governmental and non-governmental institutions on the importance of promoting sexual health education and services, including making PrEP available and covering its cost for the MSM community, in order to contain the epidemics at an early stage. Additionally, the high prevalence of recreational drug use among the sample and its possible ties with risky sexual practices require efforts to advocate harm reduction and the breaking of prejudicial punitive laws and policies that are hindering access to services, consequently deterring the health of MSM and others.
